# Effect of Heat-Killed *Lactobacillus paracasei* KW3110 Ingestion on Ocular Disorders Caused by Visual Display Terminal (VDT) Loads: A Randomized, Double-Blind, Placebo-Controlled Parallel-Group Study

**DOI:** 10.3390/nu10081058

**Published:** 2018-08-09

**Authors:** Yuji Morita, Kenta Jounai, Mika Miyake, Masaharu Inaba, Osamu Kanauchi

**Affiliations:** 1 Research Laboratories for Health Science & Food Technologies, Kirin Company, Ltd., 1-13-5 Fukuura Kanazawa-ku, Yokohama-shi, Kanagawa 236-0004, Japan; m-miyake@kirin.co.jp (M.M.); kanauchio@kirin.co.jp (O.K.); 2 Technical Development Center, Koiwai Dairy Products Co. Ltd., Sayama, Saitama 350-1321, Japan; k_jounai@koiwai-dairy.co.jp; 3 Inaba Ganka Eye Clinic, Osaka 530-0001, Japan; inaba@ganka.com

**Keywords:** *Lactobacillus paracasei* KW3110, probiotics, eye fatigue, VDT

## Abstract

Background: Visual display terminals (VDTs) emitting blue light can cause ocular disorders including eye fatigue. Some dietary constituents have been reported to be effective in improving ocular disorders while few clinical studies have been performed. We evaluated the effects of heat-killed *Lactobacillus paracasei* KW 3110 on improving ocular disorders and symptoms of eye fatigue among healthy human subjects with VDT loads. Methods: In vitro, the effect of *L. paracasei* KW3110 on blue light-induced human retinal pigment epithelial (ARPE-19) cell damage. For clinical studies, 62 healthy Japanese volunteers of 35 to 45 years of age who had experienced eye fatigue were randomized into two groups and given a placebo or *L. paracasei* KW3110-containing supplements for eight weeks. The primary endpoint was changes in VDT load-induced eye fatigue as determined by critical flicker frequency four and eight weeks after the start of supplementation. Results: In vitro, blue light-induced human retinal cell death was suppressed with the culture supernatants of cells treated with *L. paracasei* KW3110. In clinical study, the VDT load-induced reduction of critical flicker frequency tended to be milder in the *L. paracasei* KW3110 group when compared with the placebo group during the fourth week. Subgroup analysis classified by the degree of eye fatigue showed that the VDT load-induced reduction of critical flicker frequency was significantly better in the high-level eye fatigue subjects from the *L. paracasei* KW3110 group when compared with the placebo group during the fourth week (*p* = 0.020). Conclusions: *L. paracasei* KW3110 suppressed blue light-induced retinal pigment epithelial cell death. In the clinical study, ingestion of *L. paracasei* KW3110 had a potential to improve eye fatigue induced by VDT loads especially high levels of eye fatigue. However, further studies should be required to show more dependable clinical efficacy of *L. paracasei* KW3110.

## 1. Introduction

In recent years, the use of visual display terminals (VDTs) including computers, smart phones, and tablet devices has increased. Daily work performed using VDTs causes various subjective symptoms and discomfort related to eye fatigue [1,2,3,4]. The various symptoms of eye fatigue include ocular pain, blurred vision, dry eye sensation, and additional musculoskeletal complaints involving headaches, stiffness of the shoulders and neck, and low back pain [5,6,7,8,9]. Since these symptoms might lead to a lower quality of vision [10,11,12], the potential resolutions needed to mitigate these symptoms have raised concerns.

Some factors are known to cause eye fatigue [13] in which one has been intensively studied [7,14,15,16]. Continuous observations and results increased accommodations and decreased blink rates, which induce the oxidative stress and can lead to eye dryness and eye fatigue [14,17]. To prevent these problems, the effects of several dietary components such as anti-oxidants [18,19], carotenoids [20], polyphenols [21,22], and *n*-3 long-chain fatty acids [23,24,25] have been tested for improving eye functions and eye health in mice and humans.

Blue light emitted from LED displays is also a potential factor of eye fatigue. Blue light can damage retinal cells, which has been reported to be related to eye fatigue [6]. The retina is composed of not only ganglion cells and photoreceptor cells but also immune cells such as retinal macrophages. Retinal macrophages play a critical role in the response to retinal inflammation caused by blue light [26,27]. Although dietary nutrients or constituents having preventive effects on retinal inflammation have great potential in improving VDT-related eye fatigue, only a few dietary constituents have been studied [28].

Lactic acid bacteria are widely consumed as probiotics and para-probiotics to enhance gut barrier function and improve the immune system. Some strains have been reported to play other roles in humans such as preventing diarrhea, allergies, and metabolic disorders [29]. However, lactic acid bacteria have not yet been reported to mitigate blue light-induced retinal inflammation.

We previously reported that *Lactobacillus paracasei* KW3110 suppressed excessive inflammation including dermatitis in mice and humans [30,31,32,33]. In this study, in order to evaluate the preventive effects of *L. paracasei* KW3110 on blue light-induced retinal damages, we first investigated the efficacy of *L. paracasei* KW3110 on blue light-induced retinal cell damage in vitro in a human retinal cell line, ARPE-19. We then performed a randomized, double-blind, placebo-controlled parallel group study of 62 subjects using VDTs over eight weeks to evaluate the effects of micronutrients containing heat-killed *L. paracasei* KW3110 on VDT load-induced signs of ocular disorders and eye fatigue.

## 2. Materials and Methods

### 2.1. In Vitro Experiments

#### 2.1.1. Preparation of Human PBMC-Derived M2 Macrophages and Culture Media

Human peripheral blood mononuclear cells (PBMCs) from healthy donors were purchased from Lonza (Basel, Switzerland). CD14+ cells were isolated from PBMCs by using a CD14+ MicroBead kit (Miltenyi Biotec, Sunnyvale, CA, USA). To generate M2 macrophages, CD14+ cells were cultured at a density of 8.0 × 10^5^ cells/mL for 6 days by using a Human M2 Macrophage Differentiation Kit (R&D Systems, Minneapolis, MN, USA). After human PBMC-derived M2 macrophage induction, *L. paracasei* KW3110 was added at a concentration of 10 μg/mL and culturing continued for 24 h. The culture supernatants were used for interleukin-10 (IL-10) levels and the effects on blue light-induced cell death.

#### 2.1.2. Enzyme-Linked Immunosorbent Assay (ELISA)

The concentrations of human IL-10 were determined using Human Interleukin 10 ELISA Kit (BD Biosciences, San Jose, CA, USA).

#### 2.1.3. Cell Culture

The human retinal pigment epithelial (RPE) cell line, ARPE-19, was obtained from the American Type Culture Collection (ATCC, Manassas, VA, USA). The cells were cultured in Dulbecco Modified Eagle’s Medium/F12 (DMEM/F12; Gibco, Carlsbad, CA, USA) with 10% fetal calf serum (Gibco) and 100 U/mL penicillin/streptomycin (Sigma-Aldrich, St. Louis, MO, USA).

#### 2.1.4. Cell Death Analyses after Blue Light Exposure

ARPE-19 cells were pretreated with cultured supernatants from human PBMC-derived M2 macrophages stimulated with or without *L. paracasei* KW3110 at a concentration of 10 μg/mL. After 24 h cultivation, the cells were exposed to 1250 lux of blue light (LEDB-SBOXH; OptoCode Corp., Tokyo, Japan) at 470 nm for 50 min. In addition, ARPE-19 cells were also pretreated with phosphate-buffered saline (PBS) or IL-10 (10 ng/mL). After 24 h of cultivation, these cells were exposed to 1250 lux of blue light (LEDB-SBOXH, OptoCode Corp.) at 470 nm for 10 min. After 24 h of light exposure, the Hoechst 33342 stain (Dojindo, Tokyo, Japan) and propidium iodide (PI) (Dojindo) were added to the culture medium at final concentrations of 8.1 μM and 1.5 μM, respectively. PI-positive cells were determined as the ratio of PI-positive to Hoechst 33342-positive cells using an In Cell Analyzer2000 (GE Healthcare, Tokyo, Japan).

#### 2.1.5. Cellular Metabolic Activity Assay

After 1250 lux of blue light exposure, the cellular metabolic activity was measured by using a water-soluble tetrazolium salt, 2-(2-methoxy-4-nitrophenyl)-3-(4-nitrophenyl)-5-(2,4-disulfophenyl)-2*H*-tetrazolium monosodium salt (WST-8). A total of 10 µL of the Cell Counting Kit-8 reagent (CCK-8; Dojindo Laboratories, Kumamoto, Japan) was added to each well and the cells were incubated at 37 °C for 3 h. The absorbance was measured at 492 nm (reference wave length, 660 nm).

### 2.2. Clinical Study

#### 2.2.1. Ethics

This clinical study adhered to the ethical standards of the Declaration of Helsinki (revised version of 2013) and the Ethical Guidelines for Medical and Health Research Involving Human Subjects of the Ministry of Education, Culture, Sports, Science, and Technology of Japan and the Ministry of Health, Labor, and Welfare of Japan. This study obtained approval from the Ethical Committee for the Oriental Ueno Detection Center, General Incorporated Association Oriental Occupational Health Association Tokyo Branch. All subjects received the appropriate information related to the study and an informed consent was obtained in writing from each volunteer prior to enrollment in the study, which received no specific grant or funding from external organizations. The study was registered with the University Hospital Medical Information Network in Japan (UMIN) Clinical Trials Registry as UMIN000022586 and was conducted in compliance with the registered protocol.

#### 2.2.2. Subjects

**Figure 1:**
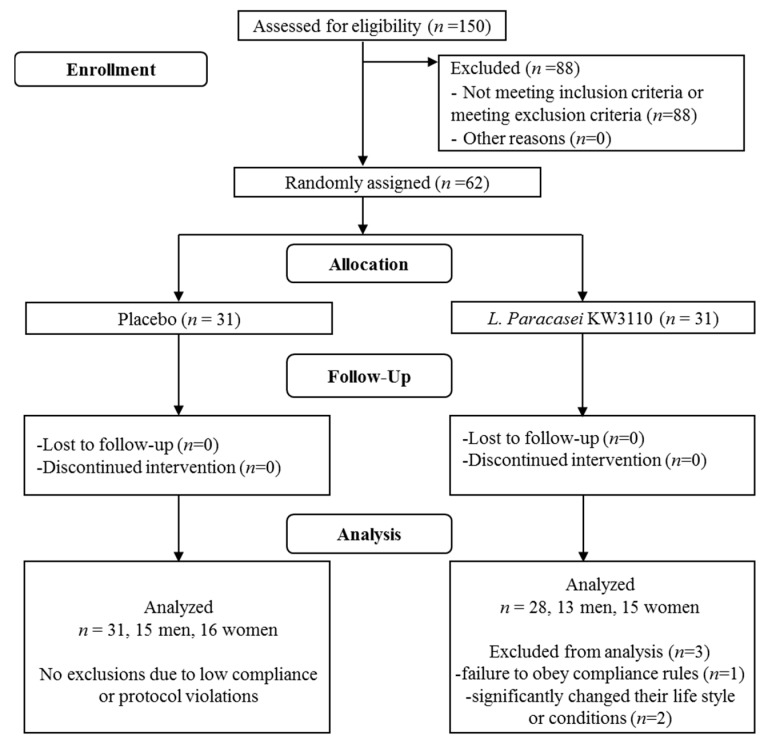
Summary flow diagram of this study.

The study enrolled healthy Japanese male and female volunteers (35 to 45 years of age) who used VDTs daily and complained about VDT-induced eye fatigue. Figure 1 illustrates the participants’ flow through the study. At first, to determine whether the candidates met the inclusion criteria, blood tests (WBC, RBC, Hb, Ht, TP, Alb, TB, PLT, total-cholesterol, TG, LDL-cholesterol, HDL-cholesterol, UN, CRE, UA, AST (GOT), ALT (GPT), γ-GT (γ-GTP), LD, Na, K, CL, and GLU) and background questionnaires including data on age, sex, medical history, usual lifestyle, and history of VDT loads were collected. The subjects were selected by the inclusion and exclusion criteria. The inclusion criteria for this study were: (1) healthy men and women aged from 35 to 45 years, (2) individuals with subjective eye fatigue, (3) individuals who used a VDT for more than 6 h per day for more than 1 year, (4) individuals providing written informed consent after receiving enough explanation of the purpose and details of the study, understanding the study well, and deciding to attend the study of their own will, and (5) approval of admission to the study by the supervising physician. In addition, subjects were excluded from the study on the basis of the exclusion criteria listed in Table 1.

#### 2.2.3. *L. paracasei* KW3110-Containing Capsules and Placebo Capsules

Subjects in the placebo group consumed hard capsules containing 200 mg non-genetically engineered cornstarch. Subjects in the *L. paracasei* KW3110 group consumed the same hard capsules containing 50 mg heat-killed *L. paracasei* KW3110 and 150 mg non-genetically engineered cornstarch for 8 weeks. The study controller confirmed that the two types of test supplements could not be distinguished based on taste, appearance, or smell.

#### 2.2.4. Study Design

The present study was designed as a randomized, double-blind, placebo-controlled, parallel group study. Both the data monitor and data analysts were blinded to the treatment assignment until the analyses were completed. This study was planned and performed by a contract research organization, TTC Co., Ltd. (Tokyo, Japan) and the data were collected at Inaba Ganka Eye Clinic (Osaka, Japan) between June 2016 and November 2016. Overall, 62 of the 150 subjects (31 male and 31 female) met the above inclusion criteria and were enrolled. The male and female subjects were randomly assigned to the placebo group (*n* = 31) or the *L. paracasei* KW3110-treated group (*n* = 31) by allocating personnel using a previously reported random number [34] in a double-blind manner. The allocation personnel were not involved in determining subject eligibility, data collection, or analysis. The sample size was calculated in accordance with a previous report that evaluated the value of CS [35]. It was calculated that, for a sample size of 29 participants in each group, statistical differences would be observed between the two groups. Therefore, we set the number of subjects as 31 in each group. The personnel managing the test foods printed a mark (A or B) on the respective outer boxes enclosing the capsules and the assignment list was kept secret by an independent party until data analyses had been completed. As such, no one knew which type of capsule each subject took. Therefore, throughout this study, the subjects, site investigator, and raters involved in this study remained blinded to each subject’s assignment. Subjects ingested one capsule of either the placebo or *L. paracasei* KW3110-containing capsule per day after breakfast with a sufficient amount of water throughout the 8-week study period. Subjects were asked to maintain their usual lifestyle during the study period. During the study, the 62 subjects were instructed to visit the hospital to have an evaluation of their eye conditions by a physician three times during week 0, week 4, and week 8 throughout the ingestion period. The evaluations of eye conditions were carried out twice on the test day. Between the two evaluations, VDT loads were carried out. The VDT loads were imposed by having the subjects play a game (Where’s Wally? in Hollywood) by using an iPad Mini (Apple Inc., Cupertino, CA, USA) for 2 h during each test session. The distance between each subject and the display was kept within 45 cm. Compliance was monitored by interview and a subject diary.

#### 2.2.5. Primary Outcome Measures

As primary outcomes, the critical flicker frequency (CFF) and eye conditions as measured by the visual analog scale (VAS) were obtained to evaluate the effect of *L. paracasei* KW3110 ingestion on eye fatigue. The CFF (Hz) was measured by using a Handy Flicker HF-II (Neitz Instruments Co., Ltd., Tokyo, Japan), according to the manufacturer’s instructions. A VAS was used to evaluate how the subjects felt about their own eye fatigue conditions. The VAS consisted of a 100 mm line drawn from “not at all” to “most” and subjects indicated the degree of each subjective eye fatigue symptom score at that moment on the line. The following criteria were evaluated by the subjects using the VAS: ocular pain, blurred vision, excess tearing, stiffness of waist or shoulders, ocular fatigue sensation, dazzled vision, double vision, frustration, stuffy head, eye redness, and headache. Constant sensitivity (CS) was also measured to evaluate the effect of *L. paracasei* KW3110 ingestion on visual function. CS was measured using a CS evaluation sheet, VTS-6000 (Vistech Inc., Hartford, CT, USA). The data were obtained before and after VDT loads at three time points, which were taken during week 0, week 4, and week 8 throughout the study.

#### 2.2.6. Secondary Outcome Measures

As secondary outcomes, near point accommodation (NPA), tear production, spherical (SPH), and subjective symptoms of dry eye were measured to evaluate the effect of *L. paracasei* KW3110 ingestion on other eye conditions. NPA and SPH were determined using an NP Accomodometer (Kowa Co., Ltd., Nagoya, Japan) or the AutorefractometerRC-5000 (Tomey Co., Nagoya, Japan), respectively. Tear production was measured using tear production measuring strips, according to the Schirmer’s test. Subjective symptoms of dry eye were evaluated using the Dry Eye-Related Quality-of-Life Score (DEQS) questionnaire [36]. The DEQS questionnaire is comprised of 15 questions including six questions assessing the ocular symptoms and nine questions assessing the effects of dry eye disease on the quality of life.

The data for NPA, tear production, and SPH were obtained before and after acute VDT loads at three time points, which were taken during week 0, week 4, and week 8 throughout the study. The data of the DEQS questionnaire was obtained before the VDT loads at three time-points, which were taken during week 0, week 4, and week 8 after the beginning of the study.

#### 2.2.7. Subgroup Analysis

Since the objective of this study was to evaluate the effects of *L. paracasei* KW3110 on VDT load-related eye disorders in the subjects who had subjective eye fatigue sensation, which is a high-level eye fatigue subgroup, the eye fatigue symptom score by VAS was greater than the average score of all subjects before the VDT load at week 0 was analyzed. Statistical analyses were performed as described in the statistical analysis section.

#### 2.2.8. Statistical Analysis

Data analyses were performed using IBM SPSS Statistical software, version 23 (IBM, Armonk, NY, USA) and Microsoft Excel 2013 (Santa Clara, CA, USA). A paired Student’s *t*-test with the Holm correction for multiple comparisons was applied to evaluate for each group the change in values from when the test capsule ingestion began (week 0). An unpaired Student’s *t*-test with a Holm correction for multiple comparisons was applied to compare differences between the two groups (i.e., placebo versus *L. paracasei* KW3110 groups) at each test point and to compare differences between the two groups for changes in values starting when the test capsule ingestion began (week 0). A *p* value of <0.05 was considered to be statistically significant. Wilcoxon signed-rank tests with Holm corrections for multiple comparisons were performed for DEQS scores in each group. The Mann-Whitney U test with the Holm correction for multiple comparisons was performed for DEQS scores between the two groups. A *p* value of <0.05 was considered to be statistically significant.

## 3. Results

### 3.1. In Vitro Experiments

#### 3.1.1. Effects of *L. paracasei* KW3110 on Human PBMC-Derived M2 Macrophage Activation

**Figure 2:**
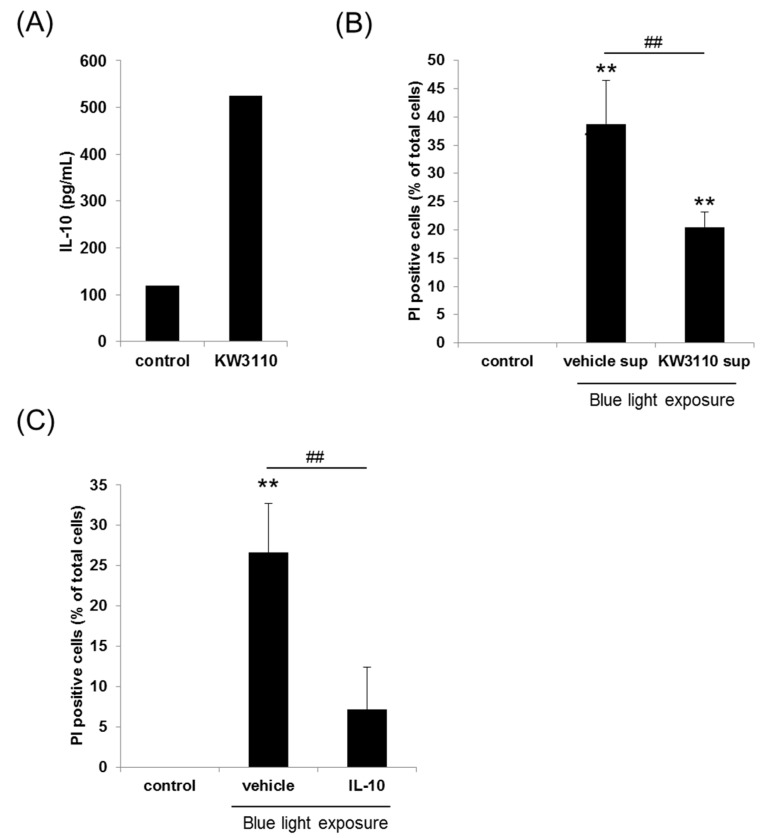
Effects of *Lactobacillus paracasei* KW3110 on cell viability under blue light exposure conditions. (**A**) *Lactobacillus paracasei* LW3110 activated human M2 macrophages. Human peripheral blood mononuclear cells (PBMC)-derived M2 macrophages were treated with 10 μg/mL *L. paracasei* KW3110 or untreated (control) and cultured for 24 h. Interleukin 10 (IL-10) concentration of the culture medium was measured by ELISAs. (**B**) The inhibitory effect of *L. paracasei* KW3110-stimulated human PBMC-derived M2 macrophage supernatants (KW3110-sup) or vehicle-treated supernatants (vehicle sup) on blue light-induced retinal cell death in ARPE-19 cells as measured by propidium iodide (PI) staining. The number of cells exhibiting PI fluorescence was counted. PI positive cells were expressed as the ratio of PI-positive to Hoechst 33342-positive cells. Assays were performed in triplicate wells. The data shows the mean ± SD for triplicate wells. ** *p* < 0.01 versus control. ## *p* < 0.01 versus vehicle supernatants [one-way analysis of variance (ANOVA) followed by Tukey’s test]. (**C**) Inhibitory effects of IL-10 on blue light-induced retinal cell death in ARPE-19 cells. The number of cells exhibiting PI fluorescence was counted. PI-positive cells were expressed as the ratio of PI-positive to Hoechst 33342-positive cells. Assays were performed in quadruplicate wells. The data show the mean ± SD for triplicate wells. ** *p* < 0.01 versus the control. ## *p* < 0.01 versus the vehicle (one-way ANOVA followed by Tukey’s test). ELISA = enzyme-linked immunosorbent assay.

To determine the effects of *L. paracasei* KW3110 on human M2 macrophage activation, human PBMC-derived M2 macrophages were treated with *L. paracasei* KW3110. IL-10 levels as an index of M2 macrophage activity were measured from culture supernatants. *L. paracasei* KW3110 induced much higher IL-10 production when compared with the control cells (Figure 2A).

#### 3.1.2. Effects of *L. paracasei* KW3110 on Blue Light-Induced Human Retinal Cell Death

We demonstrated blue light-induced human retinal pigment epithelial (ARPE-19) cell damage using a cell culture model in vitro. PI-positive dead cells, which were expressed as the ratio of PI-positive to Hoechst 33342-positive cells, were counted. To study the effect of blue light exposure toxicity on the ARPE-19 cell survival, ARPE-19 cells were incubated and exposed to 40, 50, 60, and 70 min of 1250 lux of blue light, respectively. There was a dose-dependent increase in PI-positive cells after blue light exposure (Figure S1). ARPE-19 cells that were exposed to 40 min and 50 min blue light exposure showed mild decrease in cell viability while cells that were exposed to 60 min and 70 min blue light exposure showed a marked decrease in cell viability to evaluate the effects of *L. paracasei* KW3110. Therefore, we performed further in vitro experiments under the 50 min of 1250 lux of the blue light exposure condition. Even though treatment with human PBMC-derived M2 macrophage supernatants (vehicle-sup) increased the number of PI-positive dead cells, treatment with *L. paracasei* KW3110-stimulated human PBMC-derived M2 macrophage supernatants (KW3110-sup) significantly inhibited blue light-induced cell death (Figure 2B and Figure S2). In the absence of blue light exposure, M2 macrophage supernatants, which were stimulated with or without *L. paracasei* KW3110, did not affect ARPE-19 cell survival (Figure S3). These results suggested that M2 macrophage supernatants, which were stimulated with *L. paracasei* KW3110, could suppress the blue light exposure-induced ARPE-19 cell death. To determine the inhibitory effects of KW3110-sup, we performed WST-8 assays. The results of the WST-8 assay indicated that blue light induced ARPE-19 cell death. KW3110-sup significantly inhibited blue light exposure-induced cell death (Figures S4 and S5). We also evaluated the effects of IL-10 on blue light-induced ARPE-19 cell death. Treatment with IL-10 significantly inhibited blue light-induced ARPE-19 cell death when compared with the vehicle control (Figure 2C).

### 3.2. Clinical Study

#### 3.2.1. Background Information and Baseline Characteristics of the Subjects

No adverse events related to the test supplement were reported throughout the course of the study. The flow chart of the subjects enrolled in this study is shown in Figure 1. In accordance with the inclusion and exclusion criteria, 62 (male = 29, female = 33) of 150 participants were selected to be the subjects, which were judged eligible for this study by the supervising physician, and enrolled randomly into either the placebo group or the *L. paracasei* KW3110-treated group (*n* = 31 per group, respectively), which is described in the Materials and Methods section. Three subjects in the *L. paracasei* KW3110 group were excluded from data analyses before releasing the double-blind data due to failure to obey compliance rules (one subject) or the judgement by the supervising physician that the other two subjects had significantly changed their lifestyle or conditions. Consequently, the data analyses were carried out on 59 subjects (28 subjects in the *L. paracasei* KW3110 group and 31 subjects in the placebo group). The baseline characteristics of the 59 subjects are presented in Table 2. There were no significant differences in any characteristics between the placebo and *L. paracasei* KW3110 groups.

#### 3.2.2. Ophthalmic Survey to Determine the Effects of *L. paracasei* KW3110 on VDT Load-Induced Eye Disorders

The results of the CFF tests are summarized in Table 3. As expected, VDT load-induced reductions in CFFs were observed at week 0 in both the placebo and the *L. paracasei* KW3110 group. There was no significant difference in the CFF values between the two groups. However, the variation, which is the difference of the values before and after the VDT load at each time point in the CFFs was significantly reduced at week 4 compared with week 0 only in the *L. paracasei* KW3110 group (*p* = 0.013) but not in the placebo group. Even though the changes from the values at week 0 in the CFFs were also evaluated (Table S1), there was no significant difference between the two groups.

In contrast, there was no effect of the VDT load on the variation of CS values in either of the two groups at any time points (Table S2). We could not detect significant differences in the variation ranges of CS between the two groups. Regarding the secondary outcomes such as other ophthalmic parameters, SPH, NPA, visual acuity, and the Schirmer test values, there were no significant differences in VDT load-induced variations in either of the two groups throughout the study (Table S3).

#### 3.2.3. Questionnaire Survey to Determine the Effects of *L. paracasei* KW3110 on VDT Load-Induced Subjective Eye Conditions

The subjects recorded their symptoms of eye conditions subjectively by the VAS system using a questionnaire format. Symptom scores for stiffness of the shoulders or waist and ocular fatigue sensation were significantly lower at week 4 and week 8 than at week 0 in both groups (*p* < 0.01 or *p* < 0.05, Table 4). There were no significant differences in VDT load-induced variations of the symptoms and the changes from the values at week 0 in any symptom between the two groups (Table 4 and Table S4). While the DEQS scores were also significantly lower at week 4 and week 8 than at week 0 in both groups (*p* < 0.05, Table S4), there were no significant differences in VDT load-induced variations of the DEQS scores between the two groups.

#### 3.2.4. Effects of *L. paracasei* KW3110 on VDT-Induced Eye Conditions in the Subjects Having a High-Level of Ocular Fatigue Sensation

Although the objective of this study was to evaluate the effects of *L. paracasei* KW3110 on VDT load-related eye disorders in the subjects who had subjective eye fatigue sensation. Eye fatigue symptom score levels by VAS differed among the subjects and a certain number of subjects had not been subjected to eye fatigue sensation. Therefore, we performed subgroup analysis. We analyzed the subjects with a high level of eye fatigue sensation whose ocular fatigue symptom score by VAS was greater than the average score of all subjects before VDT load during week 0. Consequently, the data analyses were carried out on 25 subjects (13 subjects in the *L. paracasei* KW3110 group and 12 subjects in the placebo group).

**Figure 3:**
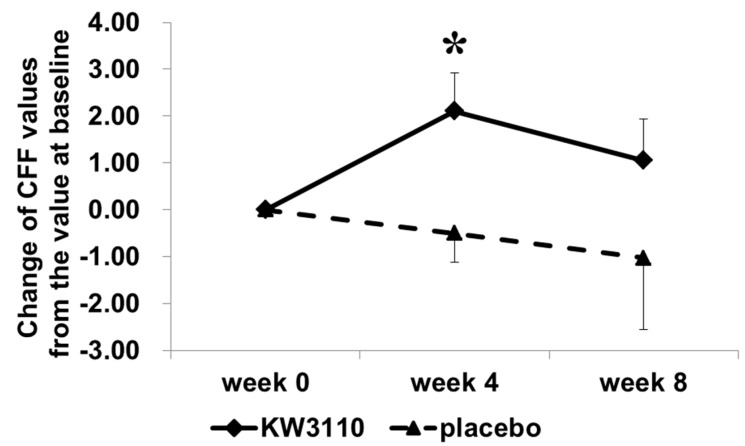
Changes of CFF values from the value during week 0 in subjects with severe ocular fatigue sensation before the VDT load. VDT = visual display terminal, CFF = critical flicker frequency. Data are calculated as the degrees of change from the values during week 0. The comparisons between two groups at each time point were performed by an unpaired Student’s *t*-test with a Holm correction for multiple comparisons (* *p* < 0.05 versus placebo). Data are shown as the means ± SE. KW3110 = *L. paracasei* KW3110-treated group, placebo = control group.

**Figure 4:**
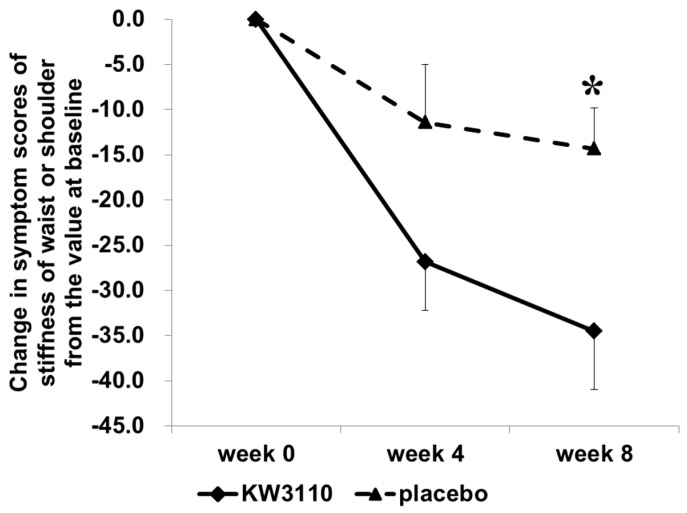
Changes in symptom scores of stiffness of the waist or shoulders from the values during week 0 in subjects with severe ocular fatigue sensation before the VDT load. VDT = visual display terminal. Data are calculated as the degrees of change from the values during week 0. The comparisons between two groups at each time point were performed by an unpaired Student’s *t*-test with a Holm correction for multiple comparisons (* *p* < 0.05 versus placebo). Data are shown as the means ± SE. KW3110 = *L. paracasei* KW3110-treated group, placebo = control group.

When the subjects with a high level of ocular fatigue sensation before VDT load were analyzed, the VDT load-induced variation in CFF as an indicator of eye fatigue was significantly reduced during week 4 compared with week 0 (Table 5) but only in the *L. paracasei* KW3110-treated group (*n* = 13, effect size, 1.087, 95% confidence interval, −0.059–3.124; *p* = 0.024) and not in the placebo group (*n* = 12). In addition, the change from the variation value at week 0 in CFF was greater in the *L. paracasei* KW3110 group than in the placebo group during week 4 (Figure 3, effect size, 1.614, 95% confidence interval, 0.519–4.686; *p* = 0.020). The subjective symptoms of eye fatigue including ocular pain, stiffness of the shoulders or waist, ocular fatigue sensation, and the stuffy head were also significantly improved in the *L. paracasei* KW3110 group (Table 6). The subjective symptom of eye redness before the VDT load was significantly reduced during week 4 in the *L. paracasei* KW3110-treated group when compared with the placebo group (Figure S6). Other subjective symptoms were not changed at any time point in both groups (data not shown). Furthermore, the changes from the values at week 0 in stiffness of the waist and/or shoulders were more reduced in the *L. paracasei* KW3110-treated group than in the placebo group during week 8 (Figure 4, effect size, −4.515; 95% confidence interval, −36.262–4.148; *p* = 0.019). This finding was inconsistent with the CFF data, which indicated that the VDT load-induced eye fatigue were more improved in the *L. paracasei* KW3110-treated group. There was no significant difference in other primary outcome (CS) and any secondary outcomes between the two groups.

## 4. Discussion

The purpose of the present study was to investigate the effects of *L. paracasei* KW3110 on VDT-induced eye disorders and eye fatigue in in vitro experiments and in a clinical trial. We demonstrated that *L. paracasei* KW3110 activated human M2 macrophages and significantly increased IL-10 production in cells. We also found that culture supernatants from cells treated with *L. paracasei* KW3110 suppressed blue light-induced retinal pigment epithelial cell death. In a randomized, double-blind, placebo-controlled parallel group study of humans with VDT load-induced eye disorders and eye fatigue, we observed that both objective parameters and subjectively quantified eye fatigue symptoms were alleviated with daily ingestion of heat-killed *L. paracasei* KW3110 supplementation. These results indicated that heat-killed *L. paracasei* KW3110 mitigated the VDT load-induced eye fatigue.

In this study, we showed that *L. paracasei* KW3110 had the potential of activating human PBMC-derived M2 macrophages and the potential of inducing the production of IL-10. In a previous study, Pam3Cys, which is a ligand for toll-like receptor 2 (TLR2), has been reported to induce IL-10 production by activating the ERK pathway in mouse dendritic cells [37]. Since lactic acid bacteria have TLR2 ligands like lipo-teichoic acid, the effect of *L. paracasei* KW3110 on IL-10 production in human PBMC-derived M2 macrophage might be mediated by the TLR2-dependent ERK pathway.

Macrophages play crucial roles in the central nervous system [38] and can be grouped into at least two subgroups including the classically activated M1 macrophages or the alternatively activated M2 macrophages [39,40]. Although M1 macrophages are pro-inflammatory and produce inflammatory cytokines such as IL-1β and tumor necrosis factor alpha (TNF-α, in contrast, M2 macrophages are associated with responses to anti-inflammatory reactions including tissue remodeling through the production of anti-inflammatory cytokines like IL-10) [41,42]. In the retina, activation of the M2 macrophage is also thought to promote retinal cell survival in several mouse models [27,43]. However, blue light exposure was reported to induce the inflammation due to the oxidative stress [44]. It is possible that M2 macrophage activation is stimulated by *L. paracasei* KW3110 and is important for the suppression of blue light-induced inflammation and human retinal pigment epithelial cell death in part through the production of IL-10 (Figure 2B,C).

VDTs including those emitting blue light may cause various visual problems and have adverse effects on the visual nervous system [6,7]. VDT load-induced visual disorders can be evaluated by objective ophthalmic parameters such as the CFF [6]. The CFF reflects neuronal impulse transmission from retinal ganglion cells to the primary visual cortex and has been used as an indicator of eye fatigue [6,45,46,47,48]. In the present study, in the *L. paracasei* KW3110-treated group with a high level of ocular fatigue sensation before VDT load, the change from the value at week 0 of VDT load-induced reduction in CFF was significantly mitigated when compared with the placebo group. These clinical results suggested that ingestion of *L. paracasei* KW3110 improved VDT load-induced eye fatigue. The initial in vitro results and a previous report also supported these clinical results. In a previous report, neural activity correlated with eye fatigue [49]. In our previous work, our group revealed that orally provided *L. paracasei* KW3110 interacted with the gut immune cells in mice and oral administration of *L. paracasei* KW3110 enhanced the cytokine levels in blood [33]. We also revealed *L. paracasei* KW3110 suppressed excessive inflammation including dermatitis in mice and humans. In this study, we showed that *L. paracasei* KW3110 could activate M2 macrophages and induce the anti-inflammatory cytokines like IL-10 (Figure 2A). We also showed that not only the M2 macrophage supernatants stimulated with *L. paracasei* KW3110, but IL-10 could significantly suppress light-induced retinal cell death (Figure 2B). Taken together, intake of *L. paracasei* KW3110 could suppress retinal inflammation in part through the anti-inflammatory cytokines in the blood, which is induced from the gut immune cells. In a previous report, a bilberry extract was also reported to protect retinal ganglion cells and also accompanied the mitigation of eye fatigue [50,51]. Therefore, the reduction of retinal cell death and inflammation might contribute to the beneficial effects of *L. paracasei* KW3110 in this study. Further clinical studies to evaluate the effects of *L. paracasei* KW3110 on inflammatory markers will be carried out to reveal the underlying mechanisms. While the relationship between retinal cell death and eye fatigue has been under investigation, the positive effects of *L. paracasei* KW3110 on VDT load-induced eye fatigue may be attributed to the inhibition of blue light-induced retinal cell death.

In our subjects, the subjective symptoms including stiffness of the waist or shoulders were mitigated by the ingestion of *L. paracasei* KW3110. The musculoskeletal discomfort including stiffness of the waist or shoulders are well-known to be associated with eye fatigue [1]. It was suggested that ingestion of *L. paracasei* KW3110 could improve musculoskeletal discomfort by reducing the symptoms of VDT load-induced eye disorders. However, the musculoskeletal symptoms were also reported to be related to blood flow [52]. Further studies are needed to delineate the mechanism underlying the effects of *L. paracasei* KW3110 on the mitigation of musculoskeletal discomfort.

In the present study, CFF and the subjective symptoms, which are the indicators of eye fatigue, were improved in the *L. paracasei* KW3110 group when compared with the placebo group. However, the improvement in CFF was significant four weeks after intake of *L. paracasei* KW3110 but was not significant during the eighth week. It is suggested that repeated eye fatigue evaluations might increase the learning effects during the eighth week, which resulted in the reduction of the difference between placebo and *L. paracasei* KW3110during the eighth week when compared with the fourth week. There was no significant difference between the *L. paracasei* KW3110 group and the placebo (control) group in other secondary outcomes including the Schirmer test results and DEQS as the indicators of dry eye. This may be because the VDT loads in this study were too mild to detect significant changes in dry eye symptoms. The results of this study were consistent with previous reports. In a previous study, VDT loads for more than eight hours could increase the risk of dry eye [53]. A previous study reported that VDT loads for two hours were too short to evaluate some parameters such as Schirmer’s test results [51]. Further studies involving longer VDT loads or brighter VDT loads might better evaluate the effect of *L. paracasei* KW3110 on VDT load-induced dry eye symptoms. However, in both CFF and VAS, which are indicators of eye fatigue, intake of *L. paracasei* KW3110 could mitigate eye fatigue when compared with the placebo group (Figure 3 and Figure 4). These results indicated that intake of *L. paracasei* KW3110 had positive effects on eye fatigue.

Since this study focused on evaluating the effects of *L. paracasei* KW3110 on VDT-induced eye disorders and eye fatigue, the clinical study enrolled only subjects who used VDT in their daily life activities. It might be necessary for the confirmation of clinical efficacy of *L. paracasei* KW3110 to carry out further studies and enrolling many more subjects. Nonetheless, there are many healthy people who have VDT work-related vision problems such as eye fatigue and the effects of *L. paracasei* KW3110 on improving VDT load-induced eye fatigue is, therefore, expected to be widely applicable.

## 5. Conclusions

In conclusion, the findings of this study indicated that *L. paracasei* KW3110 suppressed blue light-induced retinal pigment epithelial cell death in vitro and ingestion of *L. paracasei* KW3110 had positive effects for improving some objective and subjective parameters of eye disorders and eye fatigue induced by VDT loads. Further studies enrolling many more subjects should be carried out to more clearly reveal the clinical effects of *L. paracasei* KW3110 on ocular disorder including eye fatigue.

## Figures and Tables

**Table 1:** Exclusion criteria for the study.

(1)	Subjects who had amblyopia or strabismus
(2)	Subjects who were diagnosed as presbyopia [the control width was less than 2.5 diopters (D) for the dominant eye]
(3)	Subjects who had an uncorrected serious refractive error for the dominant eye
(4)	Subjects who had undergone ophthalmic surgery for the dominant eye
(5)	Subjects who had best-corrected visual acuity <1.0 for the dominant eye
(6)	Subjects who had a serious eye disease
(7)	Subjects who were under treatment for any chronic disease and/or used medicines continuously or medical supplies that were being commonly used
(8)	Subjects who had the experience of a serious disease (e.g., diabetes, liver disease, kidney disease, and/or heart disease, thyroid gland disease, adrenal gland disease, digestive system disease, and/or metabolic disorder)
(9)	Subjects who could not stop eating foods similar to the test foods and/or were taking drugs or health foods including lactic acid bacteria or Bifidobacterium
(10)	Subjects with excessive alcohol-drinking behavior
(11)	Subjects who regularly took drugs or health foods, which might have had effects on the eyes or were expected to be used during the study
(12)	Subjects who could not stop taking drugs or health foods, which might have effects on immune functions
(13)	Subjects with the possibility of drug and/or food allergy
(14)	Subjects who could not stop drinking alcoholic beverages for 2 days until the pre-check day and check days
(15)	Subjects who had a history of pollinosis
(16)	Subjects who have a tendency to get diarrhea by eating dairy products
(17)	Subjects diagnosed with drug or alcohol dependence
(18)	Subjects who and/or whose family worked in a company selling or manufacturing health foods or foods with functional claims
(19)	Subjects who could not execute a display work load test
(20)	Subjects who were participating in other clinical studies or revoked an agreement acquisition day and/or participated in other clinical studies within 3 months or who were planning to participate in other clinical studies during this study
(21)	Subjects who were pregnant, breastfeeding, planning to get pregnant, or breastfeeding during the study
(22)	Subjects who were judged as unsuitable by the physician for laboratory data, anthropometric measurements, or physical examination values
(23)	Subjects who were judged as unsuitable for the study as assessed by the background questionnaire
(24)	Subjects who were judged as unsuitable by the supervising physician for other reasons

**Table 2:** Baseline characteristics of subjects analyzed in the study.

Characteristic	KW3110	Placebo	*p* Values
	Mean ± SD	Mean ± SD	
Number of subjects (male/female)	28 (13/15)	31 (15/16)	0.880
Age (years)	40.3 ± 2.7	40.6 ± 2.8	0.651
Body weight (kg)	58.27 ± 10.29	59.34 ± 10.60	0.695
BMI (kg/m^2^)	21.48 ± 2.53	21.86 ± 2.40	0.564

Data are expressed as the mean ± standard deviation (SD) with the exception of sex. The comparisons between two groups were performed by an unpaired Student’s *t*-test except for the number of subjects (male/female). The number of subjects (male/female) was evaluated using a χ^2^ test. There was no significant difference in any parameters between the two groups. BMI = body/mass index. KW3110 = *L. paracasei* KW3110-treated group. placebo = control group.

**Table 3:** Effects of *L. paracasei* KW3110 on CFF values.

CFF (Hz)	KW3110 Group (*n* = 28)			Placebo Group (*n* = 31)		
	Week 0	Week 4	Week 8	Week 0	Week 4	Week 8
Before VDT load	39.13 ± 2.47	39.57 ± 2.95	39.98 ± 2.94	39.03 ± 2.62	39.45 ± 2.36	39.77 ± 2.83
After VDT load	37.68 ± 2.40	39.87 ± 2.52 **	39.56 ± 2.92 **	38.10 ± 2.97	39.24 ± 2.15 *	39.95 ± 2.13 *
Variation ^a^	−1.45 ± 2.38	0.30 ± 2.70 *	−0.42 ± 2.31	−0.94 ± 2.04	−0.22 ± 2.03	0.17 ± 2.97

Data are expressed as the mean ± SD. The within-group comparisons between week 0 and week 4 or week 8 were carried out using a paired Student’s *t*-test with a Holm correction for multiple comparisons (** *p* < 0.01, * *p* < 0.05). ^a^ Variation indicated the difference of the values before and after the VDT load at each point. CFF = critical flicker frequency. VDT = visual display terminal. KW3110 = *L. paracasei* KW3110-treated group, placebo = control group.

**Table 4:** The subjective symptoms of eye fatigue and related visual conditions.

Symptoms		KW3110 Group (*n* = 28)			Placebo Group (*n* = 31)		
		Week 0	Week 4	Week 8	Week 0	Week 4	Week 8
Ocular pain	Before VDT load	15.0 ± 17.9	9.3 ± 13.5	7.4 ± 14.3	12.5 ± 20.2	7.8 ± 14.3	8.9 ± 18.2
	After VDT load	28.5 ± 28.7	23.4 ± 26.4	17.6 ± 22.7	25.5 ± 26.6	20.5 ± 25.5	16.7 ± 22.3
	Variation ^a^	13.5 ± 20.8	14.1 ± 19.8	10.3 ± 16.6	13.0 ± 20.3	12.7 ± 21.4	7.8 ± 16.0
Blurred vision	Before VDT load	15.6 ± 22.0	9.8 ± 16.9	8.1 ± 14.6	13.8 ± 15.8	10.2 ± 17.1	9.5 ± 15.5
	After VDT load	25.0 ± 26.3	19.6 ± 25.4	16.7 ± 24.5	26.3 ± 26.1	20.6 ± 24.3	19.8 ± 22.9
	Variation ^a^	9.4 ± 16.4	9.8 ± 15.4	8.6 ± 17.5	12.5 ± 21.1	10.4 ± 17.8	10.4 ± 14.9
Excess tearing	Before VDT load	8.8 ± 14.5	8.9 ± 15.7	3.5 ± 5.7	8.2 ± 13.7	7.9 ± 17.0	7.3 ± 17.3
	After VDT load	16.8 ± 22.0	13.6 ± 23.8	12.9 ± 19.2	17.8 ± 23.5	13.7 ± 21.4	11.4 ± 19.8
	Variation ^a^	8.1 ± 18.8	4.7 ± 20.9	9.4 ± 15.5	9.6 ± 20.3	5.8 ± 12.8	4.1 ± 13.4
Stiffness of waist or shoulder	Before VDT load	40.9 ± 24.5	29.9 ± 22.9 *	21.1 ± 20.7 **	31.7 ± 23.7	23.9 ± 24.9 *	20.2 ± 22.0 **
	After VDT load	60.3 ± 21.8	44.3 ± 29.5 **	42.1 ± 25.5 **	48.0 ± 31.3	42.3 ± 31.4	35.5 ± 26.5 **
	Variation ^a^	19.4 ± 16.3	14.4 ± 21.2	21.0 ± 19.1	16.3 ± 25.7	18.4 ± 24.8	15.3 ± 21.5
Ocular fatigue sensation	Before VDT load	33.8 ± 25.6	23.0 ± 25.0	16.1 ± 20.6 **	27.5 ± 23.9	16.5 ± 20.5 **	15.0 ± 23.1 **
	After VDT load	56.7 ± 26.4	43.6 ± 29.7 **	37.9 ± 27.8 **	53.9 ± 26.1	42.5 ± 30.4 **	38.2 ± 26.7 **
	Variation ^a^	22.9 ± 18.3	20.6 ± 21.2	21.8 ± 23.4	26.4 ± 22.1	26.0 ± 24.2	23.2 ± 19.2
Dazzled vision	Before VDT load	7.4 ± 14.9	7.9 ± 15.9	5.8 ± 14.1	5.9 ± 9.9	5.2 ± 11.6	2.6 ± 6.0
	After VDT load	17.6 ± 20.1	12.9 ± 20.7	13.9 ± 23.1	14.9 ± 22.4	11.2 ± 21.5	9.3 ± 19.8
	Variation ^a^	10.2 ± 16.5	5.0 ± 13.8	8.2 ± 17.4	9.0 ± 19.6	6.0 ± 23.5	6.6 ± 16.9
Double vision	Before VDT load	7.1 ± 14.1	7.5 ± 15.5	5.1 ± 13.2	4.2 ± 9.1	5.4 ± 11.5	3.1 ± 6.0
	After VDT load	12.8 ± 20.1	11.8 ± 19.9	11.4 ± 22.7	11.0 ± 22.0	9.4 ± 19.9	4.0 ± 7.4
	Variation ^a^	5.7 ± 14.6	4.3 ± 12.0	6.3 ± 16.0	6.8 ± 19.5	4.0 ± 20.9	0.9 ± 4.1
Frustration	Before VDT load	11.3 ± 15.6	8.2 ± 12.0	7.4 ± 11.0	9.2 ± 16.6	9.1 ± 16.8	5.9 ± 10.2
	After VDT load	25.1 ± 27.7	21.4 ± 26.9	20.3 ± 27.1	18.7 ± 26.0	14.7 ± 17.7	14.5 ± 17.9
	Variation ^a^	13.9 ± 19.2	13.3 ± 23.2	12.9 ± 21.0	9.5 ± 29.0	5.6 ± 18.9	8.5 ± 16.2
Stuffy head	Before VDT load	18.0 ± 22.0	11.0 ± 14.3	7.8 ± 13.8 *	11.9 ± 20.7	7.9 ± 12.9	7.8 ± 15.8
	After VDT load	31.8 ± 27.8	23.5 ± 28.7	20.3 ± 25.6 *	19.3 ± 26.8	18.6 ± 28.7	17.5 ± 25.0
	Variation ^a^	13.8 ± 22.3	12.6 ± 22.8	12.5 ± 19.6	7.4 ± 15.2	10.7 ± 22.6	9.6 ± 16.3
Eye redness	Before VDT load	12.2 ± 16.2	8.6 ± 11.6	6.9 ± 12.1	13.2 ± 22.1	13.6 ± 23.7	11.4 ± 25.6
	After VDT load	14.9 ± 21.6	15.4 ± 26.0	10.8 ± 20.4	17.0 ± 23.5	13.6 ± 22.3	10.3 ± 20.5
	Variation ^a^	2.7 ± 17.2	6.9 ± 20.2	3.9 ± 16.3	3.8 ± 9.7	−0.1 ± 11.2	−1.1 ± 17.0
Headache	Before VDT load	10.0 ± 17.4	7.8 ± 11.8	4.5 ± 9.7	7.5 ± 16.9	6.9 ± 15.2	6.5 ± 15.9
	After VDT load	26.9 ± 28.4	18.9 ± 24.0	14.6 ± 21.9	18.4 ± 25.7	15.4 ± 24.6	15.5 ± 23.4
	Variation ^a^	16.9 ± 27.1	11.1 ± 20.1	10.0 ± 17.1	10.8 ± 19.5	8.4 ± 18.5	8.9 ± 15.1

Data are expressed as the mean ± SD. The within-group comparisons between week 0 and week 4 or week 8 were carried out using a paired Student’s *t*-test with a Holm correction for multiple comparisons (** *p* < 0.01, * *p* < 0.05). ^a^ Variation indicated the difference of the values before and after the VDT load at each point. VDT = visual display terminal. KW3110 = *L. paracasei* KW3110-treated group, placebo = control group.

**Table 5:** Effects of *L. paracasei* KW3110 on CFFs of subjects with severe eye fatigue.

	KW3110 Group (*n* = 13)			Placebo Group (*n* = 12)		
	Week 0	Week 4	Week 8	Week 0	Week 4	Week 8
Before VDT load	39.36 ± 2.32	39.03 ± 2.05	40.51 ± 3.10	38.75 ± 2.75	39.56 ± 2.37	40.42 ± 3.46
After VDT load	37.87 ± 2.29	39.64 ± 1.91 *	40.08 ± 2.66 *	38.33 ± 3.32	38.64 ± 2.07	38.97 ± 1.79
Variation ^a^	−1.49 ± 2.35	0.62 ± 2.18 *	−0.44 ± 2.47	−0.42 ± 2.48	−0.92 ± 1.75	−1.44 ± 3.48

Data are expressed as the mean ± SD. The within-group comparisons between week 0 and week 4 or week 8 were carried out using a paired Student’s *t*-test with a Holm correction for multiple comparisons (* *p* < 0.05). ^a^ The variation indicated the difference of the values before and after the VDT load at each point. VDT = visual display terminal, CFF = critical flicker frequency. KW3110 = *L. paracasei* KW3110-treated group, placebo = control group.

**Table 6:** Effects of *L. paracasei* KW3110 on subjective symptoms of eye fatigue and related visual conditions for subjects with severe eye fatigue.

Symptoms		KW3110 Group (*n* = 13)			Placebo Group (*n* = 12)		
		Week 0	Week 4	Week 8	Week 0	Week 4	Week 8
Ocular pain	Before VDT load	28.0 ± 18.0	7.7 ± 5.5 *	11.5 ± 19.9	25.6 ± 26.5	17.4 ± 19.0	20.6 ± 25.2
	After VDT load	49.2 ± 26.7	31.2 ± 24.8	24.5 ± 22.8	38.4 ± 30.6	35.3 ± 29.6	32.2 ± 28.1
	Variation ^a^	21.2 ± 25.9	23.5 ± 24.1	12.9 ± 16.4	12.8 ± 27.7	17.9 ± 28.6	11.6 ± 23.9
Stiffness of waist or shoulder	Before VDT load	60.8 ± 13.8	33.9 ± 21.3 **	26.2 ± 25.0 **	50.4 ± 15.8	39.0 ± 21.8	36.1 ± 25.0 **
	After VDT load	74.5 ± 13.1	51.8 ± 27.8 *	50.4 ± 26.2 **	65.6 ± 24.1	60.5 ± 22.5	51.8 ± 22.6 *
	Variation ^a^	13.7 ± 12.5	17.9 ± 26.8	24.2 ± 23.4	15.2 ± 18.5	21.5 ± 18.5	15.8 ± 14.3
Ocular fatigue sensation	Before VDT load	59.1 ± 10.1	28.3 ± 23.8 **	23.8 ± 26.0 **	53.3 ± 15.2	34.5 ± 22.5 *	35.8 ± 25.8 *
	After VDT load	74.8 ± 15.4	55.7 ± 24.7 *	50.2 ± 25.0 **	71.4 ± 17.4	62.6 ± 22.8	61.8 ± 18.7
	Variation ^a^	15.7 ± 16.0	27.4 ± 22.6	26.5 ± 27.4	18.2 ± 16.6	28.1 ± 20.5	26.0 ± 19.4
Stuffy head	Before VDT load	32.2 ± 24.6	12.3 ± 12.3 *	10.8 ± 18.2 *	21.5 ± 28.0	16.1 ± 16.6	15.8 ± 21.9
	After VDT load	43.5 ± 28.4	29.5 ± 28.2	25.1 ± 29.0	32.9 ± 30.4	31.0 ± 30.3	29.0 ± 28.2
	Variation ^a^	11.4 ± 19.2	17.2 ± 28.5	14.3 ± 20.4	11.4 ± 19.1	14.9 ± 19.8	13.2 ± 18.0

Data are expressed as the mean ± SD. The within-group comparisons between week 0 and week 4 or week 8 were carried out using a paired Student’s *t*-test with a Holm correction for multiple comparisons (** *p* < 0.01, * *p* < 0.05). ^a^ Variations indicate the difference of the values before and after the VDT load at each point. VDT = visual display terminal. KW3110 = *L. paracasei* KW3110-treated group, placebo = control group.

## Supplementary Materials

The following are available online at http://www.mdpi.com/2072-6643/10/8/1058/s1. Figure S1: Dose-dependent effects of blue light exposure on ARPE-19 cell survival. Data are shown as the mean ± SE. Figure S2: Effects of M2 macrophage supernatants, stimulated with or without *L. paracasei* KW3110, on blue light-induced ARPE-19 cell survival. Representative fluorescence microscopy images of Hoechst 33342 (blue) and propidium iodide (PI) (red) after 12 h of blue light exposure. Figure S3: Effects of M2 macrophage supernatants, stimulated with or without *L. paracasei* KW3110, on ARPE-19 cell survival in the absence of blue light exposure. Data are shown as the mean ± SE. Figure S4: Effects of M2 macrophage supernatants, which are stimulated with or without *L. paracasei* KW3110, on ARPE-19 cell survival as determined by the WST-8 assay. The inhibitory effect of *L. paracasei* KW3110-stimulated human PBMC-derived M2 macrophage supernatants (KW3110-sup) or vehicle-treated supernatants (vehicle sup) on blue light-induced retinal cell death in ARPE-19 cells, which was measured by using WST-8 assays. Assays were performed in triplicate wells. The data show the mean ± SE for triplicate wells. ** *p* < 0.01 versus control, # *p* < 0.05 versus vehicle supernatants [one-way analysis of variance (ANOVA) followed by Tukey’s test]. Figure S5: Effects of IL-10 on blue light-induced ARPE-19 cell death. Representative fluorescence microscopy images of Hoechst 33342 (blue) and propidium iodide (PI) (red) after 12 hours of blue light exposure. Figure S6: Effects of *L. paracasei* KW3110 on eye redness for subjects with severe eye fatigue. The comparisons of eye redness in visual analog scale (VAS) values at each time point were performed by an unpaired Student’s *t*-test with a Holm correction for multiple comparisons (* *p* < 0.05 versus placebo). Data are shown as the means ± SE. KW3110 = *L. paracasei* KW3110-treated group, placebo = control group. Table S1: Changes of CFF from the value at week 0. Data are expressed as the mean ± SD. ^a^ Variation indicated the difference of the values before and after the VDT load at each point. VDT = visual display terminal. CFF = critical flicker frequency. KW3110 = *L. paracasei* KW3110-treated group. placebo = control group. Table S2: Effects of *L. paracasei* KW3110 on CS values. Data are expressed as the mean ± SD. The within-group comparisons between week 0 and week 4 or week 8 were carried out using a paired Student’s *t*-test with a Holm correction for multiple comparisons (** *p* < 0.01, * *p* < 0.05). ^a^ Variation indicated the difference of the values between before and after the VDT load at each point. VDT = visual display terminal. CS = contrast sensitivity. KW3110 = *L. paracasei* KW3110-treated group. placebo = control group. Table S3: SPH, Visual acuity, NPA, Schirmer’s tests, and DEQS questionnaire values. Data are expressed as the mean ± SD. The within-group comparisons between week 0 and week 4 or week 8 were carried out using a paired Student’s *t*-test with a Holm correction for multiple comparisons (** *p* < 0.01, * *p* < 0.05). ^a^ Variation indicated the difference of the values between before and after the VDT load at each point. VDT = visual display terminal. NPA, near point accommodation. DEQS = Dry Eye-Related Quality-of-Life Score. SPH = spherical and subjective symptoms. KW3110 = *L. paracasei* KW3110-treated group. placebo = control group. Table S4: Changes of 11 subjective symptoms of eye fatigue and related visual conditions from the values at week 0. Data are expressed as the mean ± SD. The within-group comparisons between week 0 and week 4 or week 8 were carried out using a paired Student’s *t*-test with a Holm correction for multiple comparisons (** *p* < 0.01, * *p* < 0.05). ^a^ Variation indicated the difference of the values between before and after the VDT load at each point. VDT = visual display terminal. KW3110 = *L. paracasei* KW3110-treated group. placebo = control group.
