# Supplementary material for: Effect of Heat-Killed Lactobacillus paracasei KW3110 Ingestion on Ocular Disorders Caused by Visual Display Terminal (VDT) Loads: A Randomized, Double-Blind, Placebo-Controlled Parallel-Group Study
Source: Nutrients. 2018 Aug 9;10(8):1058. doi: 10.3390/nu10081058 (PMC6116181; doi:10.3390/nu10081058)

Supplementary table 1

Supplementary table 1 Change of CFF from the value at baseline

Variables		KW3110 group (n=28)		Placebo group (n=31)	
		week 4	week 8	week 4	week 8
CFF (Hz)	Before VDT load	0.44 ± 3.42	0.85 ± 2.80	0.42 ± 2.84	0.74 ± 4.10
	After VDT load	2.19 ± 2.67	1.88 ± 2.96	1.14 ± 2.70	1.85 ± 3.42
	Variation[a]	1.75 ± 3.47	1.04 ± 3.15	0.72 ± 2.88	1.11 ± 4.12

Supplementary table 2

Supplementary table 2 Effects of *L. paracasei* KW3110 on CS values

Variables		KW3110 group (n=28)			Placebo group (n=31)		
		Week 0	week 4	week 8	Week 0	week 4	week 8
CS A(1.5)	Before VDT load	5.5 ± 0.9	5.4 ± 0.6	5.4 ± 0.6	5.4 ± 0.5	5.5 ± 0.8	5.5 ± 0.6
	After VDT load	5.5 ± 0.6	5.3 ± 0.5	5.5 ± 0.6	5.2 ± 0.6	5.4 ± 0.6	5.3 ± 0.5
	Variation[a]	-0.1 ± 0.5	-0.1 ± 0.7	0.2 ± 0.7	-0.2 ± 0.6	-0.1 ± 0.8	-0.1 ± 0.7
CS B(3)	Before VDT load	6.1 ± 0.7	6.1 ± 0.4	6.1 ± 0.6	6.1 ± 0.5	6.1 ± 0.5	6.1 ± 0.6
	After VDT load	6.0 ± 0.6	6.0 ± 0.5	6.1 ± 0.6	6.0 ± 0.6	6.0 ± 0.4	6.0 ± 0.5
	Variation[a]	-0.1 ± 0.6	-0.1 ± 0.4	0.1 ± 0.7	-0.1 ± 0.6	-0.1 ± 0.6	-0.1 ± 0.6
CS C(6)	Before VDT load	5.8 ± 0.8	5.9 ± 0.5	6.0 ± 0.4	5.8 ± 0.6	6.0 ± 0.7	6.1 ± 0.7
	After VDT load	5.5 ± 0.7	5.9 ± 0.5**	6.0 ± 0.7**	5.7 ± 0.7	5.9 ± 0.5	5.8 ± 0.8
	Variation[a]	-0.3 ± 0.8	0.0 ± 0.5	0.0 ± 0.7	-0.1 ± 0.6	-0.1 ± 0.8	-0.2 ± 0.9
CS D(12)	Before VDT load	6.1 ± 0.9	6.1 ± 1.0	6.3 ± 0.7	6.1 ± 0.9	6.4 ± 0.7*	6.4 ± 0.8
	After VDT load	6.1 ± 0.8	6.1 ± 0.9	6.2 ± 0.8	5.9 ± 1.1	6.3 ± 0.7	6.2 ± 1.1
	Variation[a]	0.0 ± 0.9	0.1 ± 0.7	-0.1 ± 0.8	-0.1 ± 1.0	-0.1 ± 0.7	-0.1 ± 0.8
CS E(18)	Before VDT load	5.3 ± 1.3	5.4 ± 1.3	5.4 ± 1.2	5.6 ± 1.0	5.7 ± 1.1	5.8 ± 0.8
	After VDT load	5.3 ± 1.3	5.3 ± 0.7	5.4 ± 1.3	5.4 ± 1.1	5.7 ± 0.7	5.5 ± 1.0
	Variation[a]	0.0 ± 0.7	0.0 ± 1.0	0.0 ± 0.8	-0.2 ± 0.9	0.0 ± 1.0	-0.3 ± 1.1

Supplementary table 3

Supplementary table 3 SPH, Visual acuity, NPA, Shirmer's test, and DEQS questionaire values

Parameters		KW3110 group (n=28)			Placebo group (n=31)		
		Week 0	week 4	week 8	Week 0	week 4	week 8
SPH	Before VDT load	-3.063 ± 2.679	-3.071 ± 2.653	-3.080 ± 2.641	-2.185 ± 2.197	-2.202 ± 2.179	-2.218 ± 2.085
	After VDT load	-3.179 ± 2.749	-3.170 ± 2.641	-3.027 ± 2.650*	-2.250 ± 2.103	-2.282 ± 2.175	-2.185 ± 2.096
	Variation[a]	-0.116 ± 0.284	-0.098 ± 0.307	0.054 ± 0.267	-0.065 ± 0.288	-0.081 ± 0.395	0.032 ± 0.308
Visual acuity	Before VDT load	1.46 ± 0.11	1.43 ± 0.14	1.44 ± 0.14	1.41 ± 0.14	1.45 ± 0.11	1.44 ± 0.12
	After VDT load	1.44 ± 0.13	1.43 ± 0.15	1.41 ± 0.14	1.46 ± 0.10	1.47 ± 0.15	1.44 ± 0.12
	Variation[a]	-0.02 ± 0.11	0.00 ± 0.17	-0.03 ± 0.13	0.05 ± 0.14	0.02 ± 0.16	0.00 ± 0.13
NPA	Before VDT load	6.32 ± 2.57	6.25 ± 2.23	6.68 ± 3.16	6.23 ± 2.60	6.60 ± 2.79	6.27 ± 2.96
	After VDT load	5.87 ± 2.54	6.13 ± 2.54	6.32 ± 2.89	5.65 ± 2.25	5.92 ± 2.66	6.05 ± 2.75
	Variation[a]	-0.45 ± 1.13	-0.13 ± 1.76	-0.36 ± 1.26	-0.58 ± 1.68	-0.68 ± 1.42	-0.22 ± 0.70
Shirmer's test	Before VDT load	13.5 ± 11.6	14.2 ± 12.2	12.8 ± 10.9	12.4 ± 9.1	11.5 ± 8.4	10.3 ± 7.9
	After VDT load	13.7 ± 10.9	10.4 ± 9.9	12.5 ± 10.9	12.4 ± 9.8	9.8 ± 7.4	10.4 ± 9.0
	Variation[a]	0.3 ± 6.4	-3.8 ± 9.0	-0.3 ± 6.0	0.0 ± 7.5	-1.6 ± 8.3	0.0 ± 4.7
DEQS total score	Before VDT load	31.0 ± 20.2	22.9 ± 19.0**	20.7 ± 19.0*	28.6 ± 21.1	19.6 ± 17.1**	18.5 ± 17.3**

Supplementary table 4

Supplementary table 4

Change of 11 subjective symptoms of eye fatigue and related visual conditions from the value at baseline

Symptoms		KW3110 group (n=28)		Placebo group (n=31)	
		week 4	week 8	week 4	week 8
Ocular pain	Before VDT load	-5.8 ± 23.2	-7.6 ± 24.3	-4.7 ± 12.0	-3.6 ± 18.5
	After VDT load	-5.1 ± 24.9	-10.8 ± 29.5	-5.0 ± 20.6	-8.7 ± 25.2
	Variation[a]	0.7 ± 16.9	-3.2 ± 19.1	-0.3 ± 16.1	-5.2 ± 25.4
Blurred vision	Before VDT load	-5.8 ± 20.9	-7.5 ± 18.8	-3.6 ± 9.8	-4.3 ± 13.2
	After VDT load	-5.4 ± 26.7	-8.3 ± 23.3	-5.7 ± 24.7	-6.4 ± 21.2
	Variation[a]	0.4 ± 20.0	-0.8 ± 19.3	-2.1 ± 21.6	-2.1 ± 15.9
Excess tearing	Before VDT load	0.2 ± 19.2	-5.3 ± 14.8	-0.3 ± 14.7	-0.9 ± 19.0
	After VDT load	-3.2 ± 15.5	-3.9 ± 14.2	-4.1 ± 25.5	-6.4 ± 23.8
	Variation[a]	-3.4 ± 21.3	1.3 ± 16.0	-3.8 ± 20.8	-5.5 ± 22.5
Stiffness of waist or shoulder	Before VDT load	-11.0 ± 24.4	-19.9 ± 22.6	-7.8± 17.8	-11.5 ± 14.9
	After VDT load	-16.0 ± 23.8	-18.2 ± 20.0	-5.7 ± 21.1	-12.5 ± 19.6
	Variation[a]	-5.0 ± 25.3	1.7 ± 20.6	2.1 ± 18.6	-1.0 ± 19.5
Ocular fatigue sensation	Before VDT load	-10.8 ± 28.8	-17.7 ± 24.9	-11.0 ± 16.2	-12.5 ± 15.2
	After VDT load	-13.1 ± 23.9	-18.8 ± 22.5	-11.5 ± 20.8	-15.8 ± 16.0
	Variation[a]	-2.3 ± 27.6	-1.1 ± 25.5	-0.4 ± 20.4	-3.2 ± 19.8
Dazzled vision	Before VDT load	0.6 ± 16.2	-1.6 ± 12.5	-0.7 ± 11.9	-3.3 ± 9.0
	After VDT load	-4.6 ± 19.3	-3.6 ± 17.8	-3.7 ± 12.7	-5.6 ± 17.7
	Variation[a]	-5.2 ± 18.6	-2.0 ± 13.3	-3.0 ± 18.0	-2.4 ± 15.4
Double vision	Before VDT load	0.4 ± 15.2	-2.0 ± 10.7	1.2 ± 11.2	-1.1 ± 9.1
	After VDT load	-1.0 ± 14.3	-1.4 ± 15.7	-1.6 ± 12.6	-7.0 ± 19.9
	Variation[a]	-1.4 ± 15.9	0.6 ± 14.5	-2.8 ± 15.9	-5.9 ± 19.0
Frustration	Before VDT load	-3.1 ± 16.7	-3.9 ± 14.4	-0.1 ± 19.4	-3.3 ± 16.4
	After VDT load	-3.7 ± 20.3	-4.9 ± 14.9	-4.0 ± 23.8	-4.2 ± 17.5
	Variation[a]	-0.6 ± 22.5	-1.0 ± 16.4	-3.9 ± 30.5	-0.9 ± 22.2
Stuffy head	Before VDT load	-7.0 ± 25.4	-10.3 ± 22.3	-4.0 ± 13.7	-4.1 ± 14.7
	After VDT load	-8.3 ± 26.1	-11.6 ± 23.2	-0.6 ± 26.8	-1.8 ± 22.8
	Variation[a]	-1.3 ± 21.7	-1.3 ± 16.8	3.4 ± 28.7	2.3 ± 22.7
Eye redness	Before VDT load	-3.6 ± 17.1	-5.3 ± 15.3	0.5 ± 11.1	-1.8 ± 15.5
	After VDT load	0.5 ± 17.9	-4.1 ± 15.4	-3.4 ± 15.1	-6.6 ± 18.0
	Variation[a]	4.2 ± 19.3	1.2 ± 14.0	-3.8 ± 12.8	-4.9 ± 16.2
Headache	Before VDT load	-2.2 ± 20.9	-5.5 ± 19.8	-0.6 ± 13.4	-1.0 ± 15.0
	After VDT load	-8.0 ± 26.8	-12.3 ± 27.9	-3.0 ± 18.8	-2.9 ± 16.6
	Variation[a]	-5.8 ± 28.1	-6.9 ± 22.3	-2.4 ± 24.0	-1.9 ± 19.4

Supplementary Figure 1

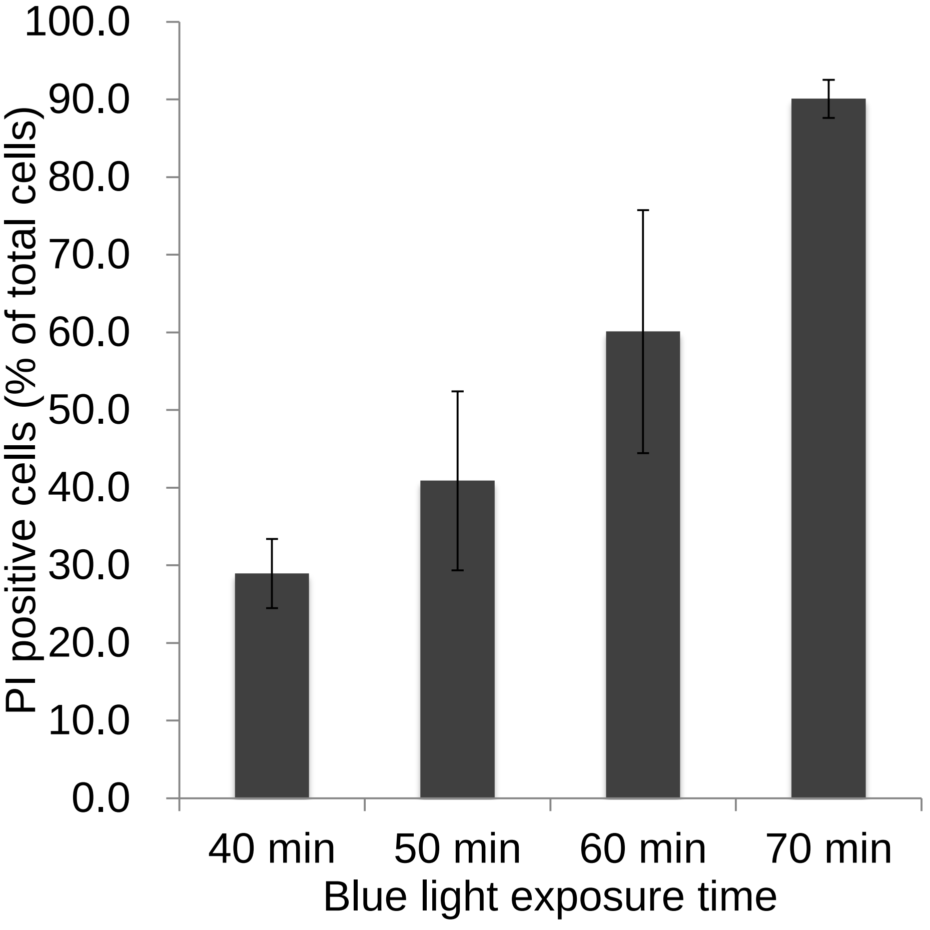

Supplementary Figure 2

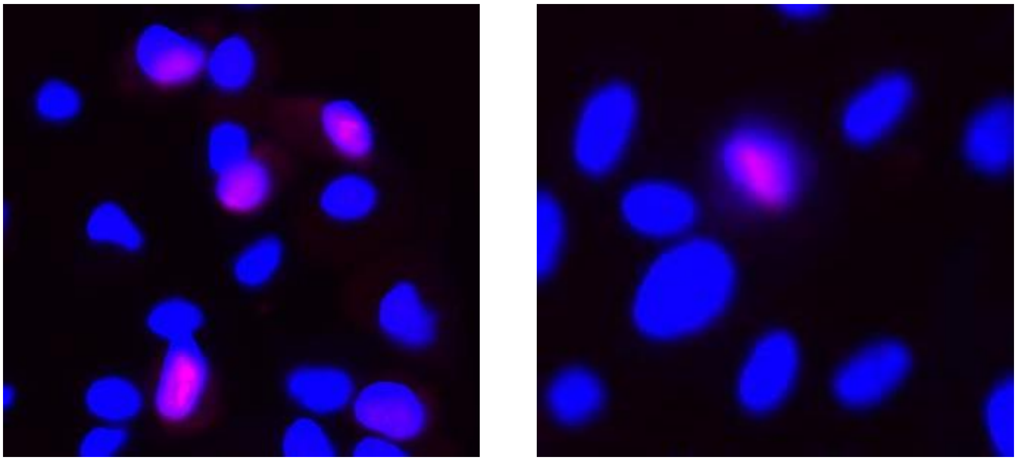

Vehicle sup KW3110 sup

Supplementary Figure 3

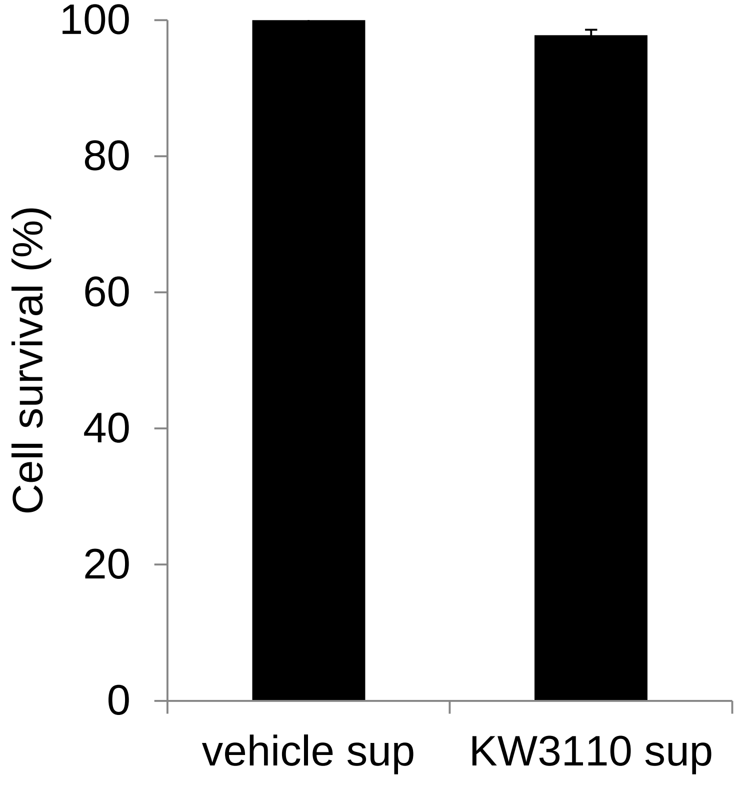

Supplementary Figure 4

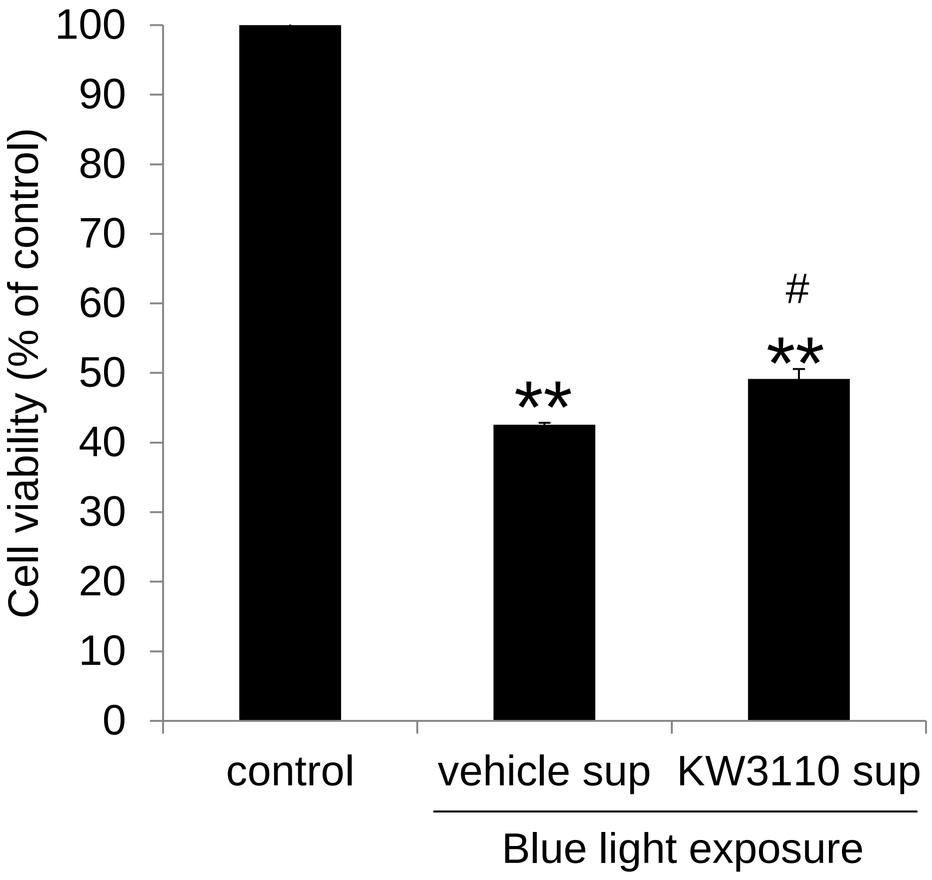

Supplementary Figure 5

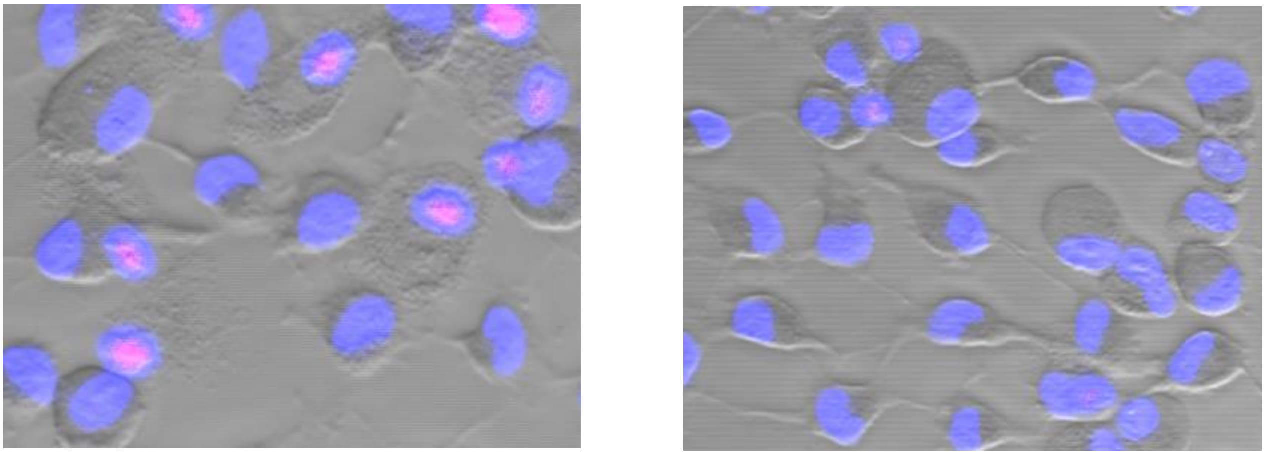

Vehicle IL-10

Supplementary figure 6

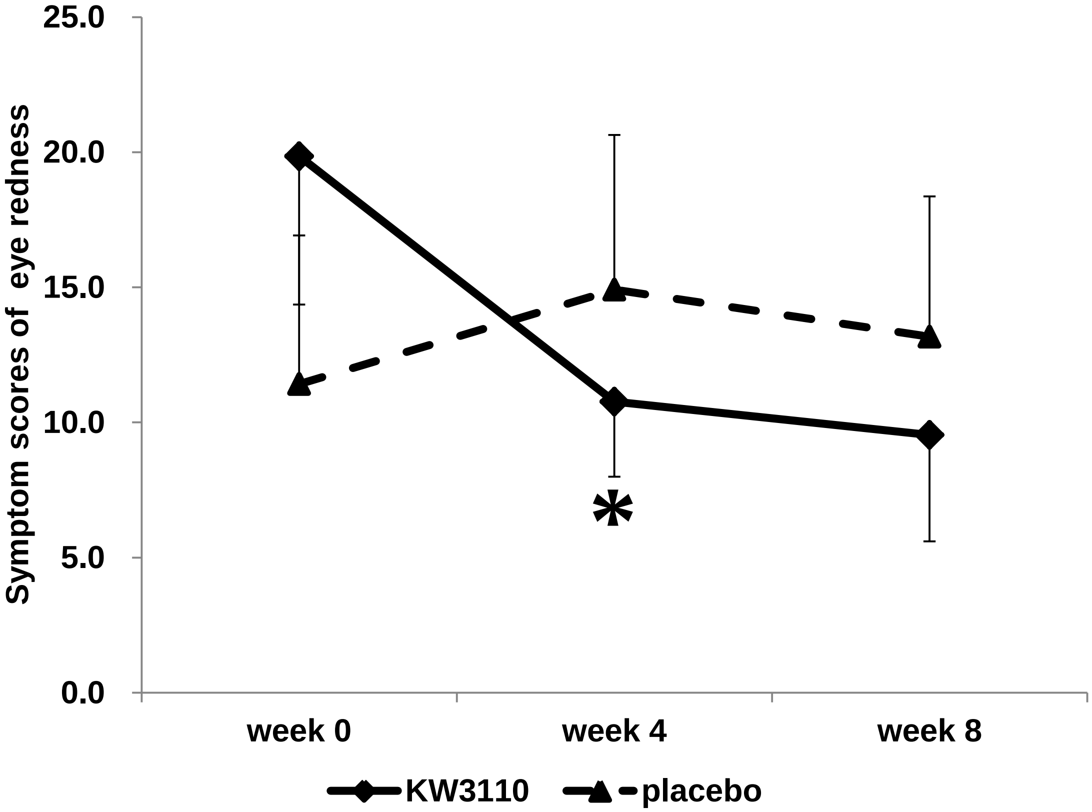

Supplement: Supplementary file 1 [file nutrients-10-01058-s001.pdf]
